# AML under the Scope: Current Strategies and Treatment Involving FLT3 Inhibitors and Venetoclax-Based Regimens

**DOI:** 10.3390/ijms242115849

**Published:** 2023-10-31

**Authors:** Szymon Milnerowicz, Julia Milnerowicz, Paulina Skowera, Magdalena Stelmach, Monika Lejman

**Affiliations:** 1 Student Scientific Society, Laboratory of Genetic Diagnostics, Medical University of Lublin, 20-093 Lublin, Poland; 56517@student.umlub.pl (S.M.); 56511@student.umlub.pl (J.M.); 2 Independent Laboratory of Genetic Diagnostics, Medical University of Lublin, 20-093 Lublin, Poland; paulina.skowera@uszd.lublin.pl (P.S.); magdalena.stelmach@uszd.lublin.pl (M.S.)

**Keywords:** acute myeloid leukemia (AML), tyrosine kinase inhibitors (TKI), FLT3 (FMS-like tyrosine kinase-3) inhibitors, FLT3-internal tandem duplication (ITD), BCL-2 (B-cell lymphoma-2) inhibitors

## Abstract

Acute myeloid leukemia (AML) is a disease that mainly affects elderly patients who are more often unfit for intensive chemotherapy (median age of diagnosis is 68). The regimens, including venetoclax, a highly specific BCL-2 (B-cell lymphoma-2) inhibitor, are a common alternative because of their safer profile and fewer side effects. However, the resistance phenomenon of leukemic cells necessitates the search for drugs that would help to overcome the resistance and improve treatment outcomes. One of the resistance mechanisms takes place through the upregulation of MCL-1 and BCL-XL, preventing BAX/BAK-driven MOMP (mitochondrial outer membrane permeabilization), thus stopping the apoptosis process. Possible partners for BCL-2 inhibitors may include inhibitors from the FLT3i (FMS-like tyrosine kinase-3 inhibitor) group. They resensitize cancer cells through the downregulation of MCL-1 expression in the *FLT3* mutated cells, resulting in the stronger efficacy of BCL-2 inhibitors. Also, they provide an additional pathway for targeting the clonal cell. Both preclinical and clinical data suggest that the combination might show a synergistic effect and improve patients’ outcomes. The aim of this review is to determine whether the combination of venetoclax and FLT3 inhibitors can impact the therapeutic approaches and what other agents they can be combined with.

## 1. Introduction

Acute myeloid leukemia (AML) refers to a large and heterogeneous category of clinically aggressive hematologic neoplasms. AML originates from a series of genetic changes in a hematopoietic precursor cell, which results in an accumulation of myeloid blasts in bone marrow, blood, or other tissues.

AML is the most prevalent type of leukemia among adult patients and is expected to account for approximately 80% of all bone marrow cancer cases in 2023, with an estimated incidence of 20,380 new cases [1,2]. The mortality rate of AML in the general population has increased from 2.21 to 2.67 deaths per 100,000 population between 1990 and 2020. Simultaneously, there has been an augmentation in the incidence rate of AML, advancing from 3.00 to 3.90 new cases per 100,000 population over the same temporal span. The observed escalation in incidence and concomitant mortality may be attributed to improved detection rates, which have enhanced the capacity to diagnose AML at earlier stages, subsequently influencing the statistical landscape. Furthermore, an increasing prevalence of obesity and an aging demographic profile are both established risk factors of AML and are associated with worse outcomes [3,4]. However, among individuals under 15 years of age, there has been a decrease in mortality from 0.20 to 0.17 deaths per 100,000 population, accompanied by an increase in incidence from 0.59 to 0.80 per 100,000 population between 1990 and 2020 [3]. The reduction in mortality within this age group may be associated with higher doses of anthracycline and improved supportive care. Additionally, the development of hematopoietic stem cell transplantation and second-line salvage chemotherapy may have contributed to improved outcomes. Conversely, the observed increase in incidence among individuals under 15 years of age may be linked to demographic shifts. Notably, an elevated parental age, particularly in fathers, has been associated with an increased risk of pediatric AML, thereby influencing the rising incidence rates [5].

Given the escalating incidence and mortality rates in the AML patient population, there is an imperative to discover novel therapeutic approaches to improve patient outcomes. The emergence of advanced molecular and cytogenetic diagnostic tools offers new opportunities to target specific mutations and variants of this heterogeneous disease, which may hold promise for the future of AML therapy [6].

This review focuses on the potential treatment strategies for AML involving a combination of FMS-like tyrosine kinase-3 (FLT3) inhibitors and B-cell lymphoma 2 (BCL-2) inhibitors and potential partners for this combination.

## 2. Results

### 2.1. BCL-2 Inhibitors

The BCL-2 proteins family is a group of 3 types of proteins: (1) the anti-apoptotic agents (e.g., BCL-2, BCL-XL, MCL-1, BCL-2 and A1), (2) pro-apoptotic effector proteins (BAK, BAX, BOK and tBID) and (3) the BH3-only proteins, which can be subdivided into sensitizers (e.g., BAD, NOXA, BIK) and activators (BID, PUMA, BIM) [7,8,9]. These factors from the BCL-2 family play a crucial role in the regulation of the intrinsic pathway of apoptosis. In the presence of cellular stress (DNA damage, uncontrolled proliferation, etc.), the BH3-only proteins activate the effector proteins, causing mitochondrial outer membrane permeabilization (MOMP). It results in an efflux of pro-apoptogenic factors like cytochrome c and SMAC, which leads to an activation of caspases and the death of a cell [10,11].

A group of BH3-mimetics, the on-target and specific inhibitors of the BCL-2 protein family, consists of several molecules. ABT-737 and navitoclax were the first to be discovered and held promise for revolution in targeted therapy for many malignancies. However, after conducting clinical trials, both of the inhibitors failed to meet the expectations, either due to adverse side effects like thrombocytopenia or because of poor bioavailability [12,13]. The side effects caused by the BCL-2 pan-inhibitors were the result of inhibition of BCL-XL, an important guardian protein that plays a key role in platelet survival. Nonetheless, the efficacy of navitoclax in trials initiated the development of yet another BH3-mimetic [14].

#### 2.1.1. Mechanism of Action of Venetoclax

It was hypothesized that selective targeting of only BCL-2 protein and sparing the BCL-XL and MCL-1 could overcome the side effects caused by navitoclax. The first BCL-2 inhibitor (BCL-2i) approved by the FDA is venetoclax, also referred to as ABT-199. It is a highly specific BH3-mimetic that binds to the BCL-2 protein groove and has very low affinity to BCL-XL [15]. Upon connection, venetoclax displaces the BCL-2 bound BIM, allowing it to activate the effector proteins BAK and/or BAX [16]. It results in the BAK/BAX-driven release of cytochrome c and apoptosis of the cell. The rapid and strong effects of venetoclax were rather surprising to researchers. Inhibition of BCL-2 alone was suspected to face an obstacle due to the possible binding of BIM to other guardian proteins, which would not result in cell apoptosis. Despite that, venetoclax shows strong activity, suggesting that many malignancies are dependent on pro-survival BCL-2 protein solely [8,12].

The reason why venetoclax targets mainly cancer cells can be explained by “priming”. It is a phenomenon in which BCL-2 molecules present on the mitochondrial outer membrane are largely occupied by pro-apoptotic proteins. A BH3-mimetic can displace large quantities of apoptotic activators, resulting in MOMP. Cells with small amounts of prebound proapoptotic factors show lesser response to BH3-mimetics and survive the process [12,17]. A primed state is more common in cancer cells than in healthy ones. However, the assay called BH3 profiling identifies whether a certain cell is in a “primed by death” state. This technique might be used in the determination of resistance or susceptibility to a certain treatment [18].

#### 2.1.2. Clinical Data for Venetoclax

As for now, venetoclax (VEN) in combination with either HMAs (hypo-methylating agents) or LDAC (low-dose cytarabine) is approved by the FDA for use in previously untreated patients with AML who are 75 years or older or who have other comorbidities precluding intensive induction therapy [19].

Venetoclax, in combination with azacitidine or decitabine, shows significant activity resulting in 48–67% complete remission (CR) + complete remission with incomplete count recovery (CRi) and its median duration of 11.3 months. The median overall survival (OS) was 7.2–17.5 months [20,21]. However, when used as a single agent in relapse/refractory (R/R) patients, venetoclax demonstrated only 19% CR/CRi [22]. On the other hand, the real-world data shows that the overall response rate was 55%, and median overall survival was 13 months in patients treated with VEN/HMA [23].

The addition of venetoclax to azatadine in treatment of *FLT3* mutated patients resulted in 67% CRc (Composite Complete Remission) and 12.5 months of median OS vs. 36% CRc and 8.6 months of median OS in azatadine-only group [24]. In this trial, both *FLT3*-mut and *FLT3*-WT (wild-type) patients treated with VEN+AZA achieved a remission rate of 67%.

Venetoclax can be used not only in conjunction with lower-intensity protocols but also as a component of intensive chemotherapy. The most popular “7+3” regimen consisting of daunorubicin and cytarabine allows 60–80% of patients to reach CR and 61% to achieve the Minimal Residual Disease-negative (MRD-negative) state, meaning that they achieved CR with at most one malignant cell detected among 1000 normal cells. [25,26]. The addition of venetoclax to the daunorubicin and cytarabine resulted in 91% CR after one cycle and MRD-negativity in 97% of those with complete response. The study was conducted on a population of 33 adults ranging from 18 to 60 years of age with de novo AML [27].

However, another study evaluated the efficacy and safety profile of induction chemotherapy with venetoclax, with a modified regimen based on daunorubicin and cytarabine in a “2+6” model in order to reduce the myelosuppression period. With a European Leukemia Network (ELN) prognostic group in 2022, the patients were classified as at adverse risk (45.2%), intermediate risk (14.3%), and favorable risk (40.5%). The outcomes were similar to those from previous studies (CRc 90.5% and MRD-negativity 87.9%), but the duration of complications was reduced. The median recovery time for neutrophils and platelets was 13 and 12 days, respectively [28].

Another phase 1b/2 study evaluating treatment outcomes for the FLAG–IDA + venetoclax regimen in newly diagnosed AML patients found that 88% of subjects attained CRc and 92% of those achieved MRD-negativity. Moreover, results presented at the 2021 Society of Hematologic Oncology suggest there is no significant difference in event-free survival (EFS) or OS with the use of hematopoietic stem cell transplantation (HSCT) in a group treated with FLAG–IDA + venetoclax [29,30]. In a real-world data analysis conducted on mostly R/R AML patients (96% R/R AML), a CRc was achieved in 72% of cases [31].

A post-hoc propensity-score matched analysis showed that intensive chemotherapy (IC) with the addition of venetoclax, compared to standard IC, resulted in improved event-free survival and, in ELN intermediate and adverse risk groups, higher overall survival. However, the most prominent effect concerns the adverse risk group. Moreover, the MRD-negative composite complete remission was 86% vs. 61% in IC + VEN and IC alone, respectively [26].

Therapies using venetoclax are associated with the occurrence of side effects, including grade 3/4. In the lower-intensity regimens with the addition of venetoclax, the most common grade 3/4 adverse events were infections and cytopenias. The febrile neutropenia occurs in 47%, grade 3/4 decrease of white blood cells in 42%, grade 3/4 anemia in 28%, and grade 3/4 thrombocytopenia in 33% [32]. In the case of IC + venetoclax treatment in newly diagnosed AML, the grade 3/4 adverse events included febrile neutropenia (39–55%), pneumonia (24–28%), bacteremia (21%) and sepsis (10%) [29,30,33]. Bacteremia is more common in R/R AML than newly diagnosed AML (46% vs. 21%) [33].

Although most of the studies on venetoclax involve adult patient populations, there are studies suggesting good efficacy and high safety profile of BCL-2 inhibitors in pediatric populations. However, venetoclax is mainly tested in R/R AML settings. The results from a phase 1 study are encouraging, with a CR/CRi in 70% of patients and 71% of MRD-negative CRc patients. However, all of the patients harboring the *FLT3* mutation (*n* = 5) did not respond to the treatment. Nonetheless, the safety profile of venetoclax is similar to the one in the adult population and can be incorporated into various regimens. The efficiency and long-term outcomes are yet to be discovered, but there are several ongoing clinical trials that will give us further information and a better understanding of BCL-2 inhibitor activity in this age group (NCT05183035, NCT04161885, NCT05317403) [34,35].

#### 2.1.3. Mechanisms of Venetoclax Resistance

**Figure 1:**
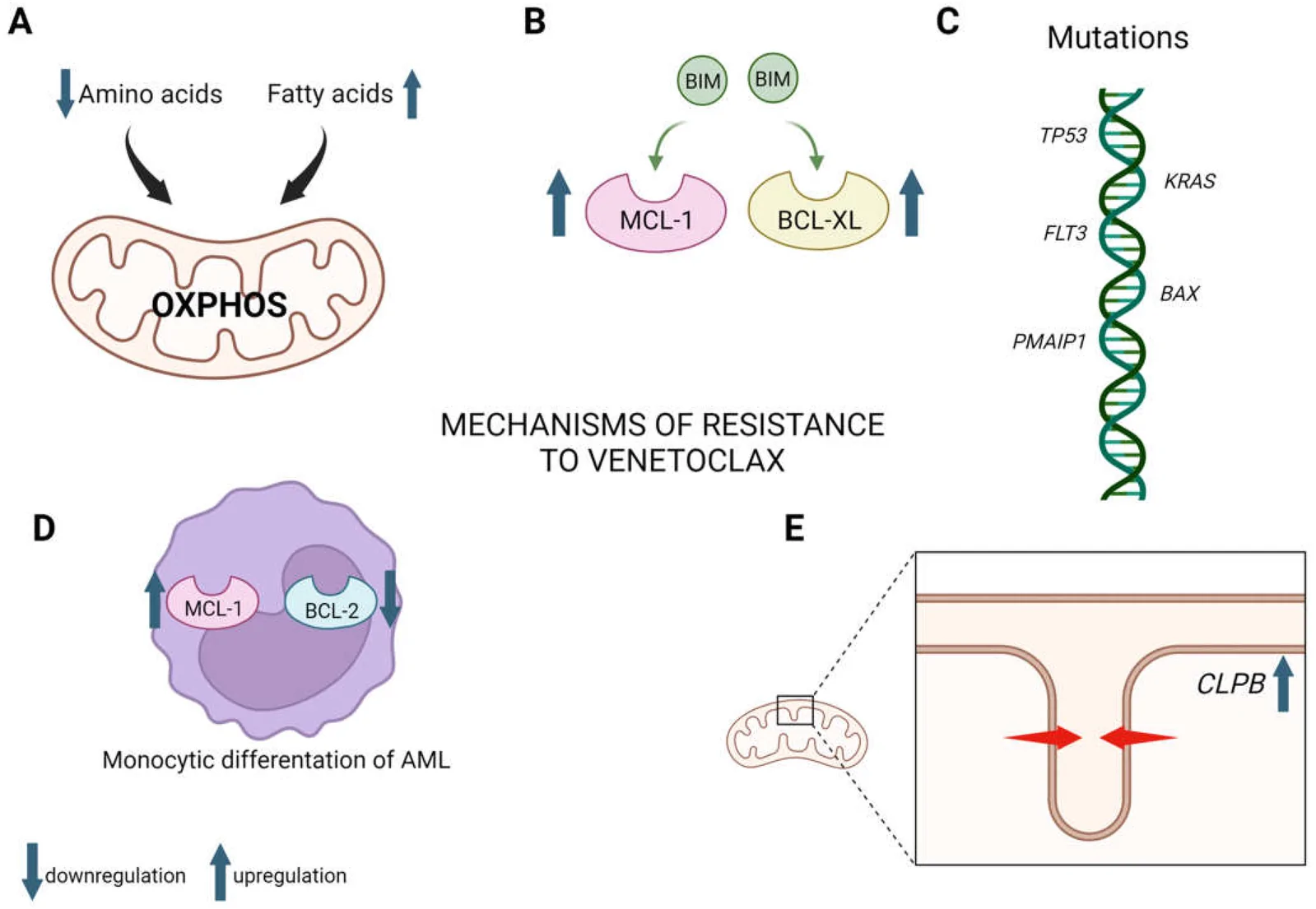
Mechanisms of resistance to venetoclax, including (**A**) alterations in energy metabolism, shifting from amino acids towards fatty acids; (**B**) upregulation of MCL-1 and BCL-XL; (**C**) mutations conferring resistance; (**D**) monocytic differentiation linked with downregulation of BCL2 and upregulation of MCL-1; (**E**): narrowing of mitochondrial cristae lumen and overexpression of CLPB. Created with BioRender.com (accessed on 17 August 2023).

Mechanisms of resistance to venetoclax have not been fully discovered yet, but there are several proposed pathways that can influence the response to BCL-2 inhibitor treatment. The upregulation of MCL-1 and BCL-XL expression exhibited by the AML cells antagonizes the mechanism of action of venetoclax by binding to BIM, thus not allowing it to induce MOMP (Figure 1B). This mechanism is considered to be the main in venetoclax resistance. It is caused by alterations in the signaling pathways like NF-κB, MAPK/ERK, PI3K/AKT, JAK/STAT, or FOXM1–AKT cycle. Those alterations can be associated with mutations in *FLT3* or *KRAS* (Figure 1C). Also, primary AML cells with monocyte phenotype were associated with decreased expression of BCL-2 and increased expression of MCL-1 (Figure 1D). Another resistance mechanism is due to altered mitochondrion structure, specifically the narrowing of the mitochondrial cristae lumen in cell lines with higher expression of mitochondrial chaperone protein CLPB (Figure 1E). The resistance to BCL-2 inhibitors might also be mediated by alterations in the metabolism. Oxidative phosphorylation (OXPHOS) is an essential source of energy for AML leukemia stem cells (LSCs). While LSCs predominantly rely upon the metabolism of amino acids for their energy requirements, they possess the capacity to circumvent this dependency by augmenting their utilization of fatty acid metabolism. This adaptation subsequently leads to a significant reduction in the sensitivity of these cells to venetoclax (Figure 1A) [36]. Additionally, increased levels of nicotinamide have been observed in R/R LSCs in comparison to de novo LSCs. Nicotinamide is metabolized to NAD+, an important piece in energy metabolism. This leads to increased overall energy metabolism, thus resulting in resistance [37,38]. Resistance may also be mediated by inactivating mutation in *BAX, PMAIP1*, and tumor suppressor gene *TP53* [39,40,41,42]. However, the mediators of apoptosis combinatorial score (MAC-score) can predict response to VEN/AZA treatment by measuring the proportion of BCL-2, MCL-1, and BCL-XL expressed proteins in the leukemic stem cells [43]. Additionally, targeting these proteins not only allows for re-sensitizing the cells to venetoclax but also makes it possible to delay or even prevent the emergence of resistance [44].

### 2.2. FLT3

FMS-like tyrosine kinase-3 (FLT3) is one of many human tyrosine kinases [45,46]. Tyrosine kinases belong to a group of enzymes that catalyze the phosphorylation of select tyrosine residues in target proteins using ATP. Due to this action, tyrosine kinases are valid mediators of the signaling cascade, determining pivotal roles in various biological processes such as growth, metabolism, differentiation, and apoptosis, which occur as a response to internal or external stimuli. The following classified subtypes of tyrosine kinases are distinguished: receptor tyrosine kinases (RTK) and non-receptor tyrosine kinases (NRTK). The RTKs are not exclusively enzymes with the activity of kinase but also cell surface transmembrane receptors [46]. There are 58 known human RTKs, and FLT3 is one of them [47]. FLT3 is of great importance in hematopoiesis; under normal conditions, it is expressed in bone marrow stem cells where the FLT3 ligand (FLT3L) activates it. FLT3 is essential for hematopoietic cell proliferation and maturation [48]. Its molecular structure as an RTK consists of an extracellular receptor part, a short α-helical transmembrane part, and an intracellular part. The receptor part is built of five immunoglobulin-like (IGL) domains: extracellular, juxtamembrane, tyrosine kinase (TK), kinase insertion, and a C-terminal intracellular domain [47,49]. During regular conditions, FLT3 is activated by the attachment of a hematopoietic growth factor, FLT3-ligand, to IGL domains D2 and D3. A ligand dimer binds to the receptor, leading to its self-dimerization. This dimerization of FLT3 converts the signal to the intracellular part, where the FLT3 kinase domains bind to and activate each other by phosphorylation [47]. The gene responsible for encoding FLT3 in humans is located at 13q12.2, and it exhibits broad expression in the spleen, lymph nodes, bone marrow, appendix, and, to a lesser extent, in some other tissues. Since the activity of FLT3 is strongly connected with marrow stromal cells and hematopoietic cells, there should be no surprise that its mutation can be connected with AML pathophysiology.

#### FLT3 Mutations

The molecular pathogenesis of AML is a complex process, and it can be challenging to differentiate between founder and driver mutations, especially with the increased utilization of genetic testing for other various indications [49,50].

While certain mutations (e.g., *DNMT3A*, *TET2*, and *ASXL1*) are frequently observed in clonal hematopoiesis and seem to occur relatively early in leukemogenesis, others tend to emerge later during the progression of leukemia, they include mutations in *FLT3*, *NRAS*, and *RUNX1* [51].

Approximately 30% of newly diagnosed AML ensues from mutations in *FLT3,* which are divided into two classes: internal tandem duplications (*FLT3*–ITD)—which involve the juxtamembrane domain, and the second class: tyrosine kinase domain mutations (*FLT3*–TKD) [48,52,53]. The percentage distribution among the *FLT3* mutations is: about 25% of adults with AML will have *FLT3*–ITD, and about 10% of adult patients will have *FLT3*–TKD point mutations or deletions [49].

*FLT3*–ITD mutated AML displays heterogeneity, which can be attributed to variations in ITD length, insertion site, mutant-to-wild type allele ratio (AR), overall karyotype, and co-occurring mutations, particularly *NPM1* [45]. Regardless of other factors, the presence of *FLT3*–ITD mutation alone in patients newly diagnosed is associated with unfavorable outcomes in terms of relapse-free (RFS) and overall survival (OS) compared to patients with wild-type *FLT3* (WT-*FLT3*) [45,49,53]. It should be pointed out that until recently, the impact of an *FLT3* mutation on the prognosis of patients struggling with AML was considered to be dependent on the ratio of mutated to wild-type allele [54]. However, according to the 2022 ELN recommendations, the *FLT3*–ITD allelic ratio is not taken into consideration in the risk classification. At the moment, AML coexisting with *FLT3*–ITD is classified as an intermediate-risk group, regardless of the allelic ratio or concurrent presence of *NPM1* mutations [51]. Despite achieving similar response rates to traditional chemotherapy, patients with *FLT3*-mutated AML have a higher likelihood of relapse than patients with WT-*FLT3* AML, even after undergoing allogeneic hematopoietic stem cell transplantation (HSCT) [49].

### 2.3. FLT3 Inhibitors

The FMS-like tyrosine kinase inhibitors (FLT3i) are a group of medications that belong to the family of tyrosine kinase inhibitors (TKI), which show promise as targeted therapeutics in the treatment of AML. The family of TKI comprises molecules that possess the capability to inhibit either cytosolic or receptor tyrosine kinases. The inhibition occurs via several possible paths: direct competition for ATP binding to the tyrosine kinase, allosteric inhibition of the tyrosine kinase, inhibition of ligand binding to receptor tyrosine kinases, inhibition of tyrosine kinase interaction with other proteins, or destabilization of the tyrosine kinase [55]. FLT3 inhibitors promote terminal differentiation of *FLT3*-mutant myeloblasts and cause direct cytotoxicity to them [56]. Given the high frequency of *FLT3* mutations in AML and their correlation with poor prognosis, in recent years, extensive research focusing on the development of targeted therapies was conducted, aiming to enhance patient outcomes. Due to these studies, FLT3i can be categorized using two classification systems: first- and second-generation based on their specificity, and Type I and II inhibitors based on their mechanism of action [45,57]. Each FLT3i inhibits receptor autophosphorylation and downstream signaling activation by interacting with the ATP-binding site of the intracellular tyrosine kinase domain. Type 1 inhibitors are active against both ITD and TKD mutations, while Type 2 inhibitors are only effective against ITD mutations [45]. Type 1 inhibitors bind to both active and inactive conformations of the receptor, while type 2 inhibitors (sorafenib and quizartinib) interact with the hydrophobic region adjoining the ATP-binding site, which is available only in the non-active state [58,59]. The FLT3 inhibitors of the first generation display broad-spectrum kinase targeting, leading to various off-target effects [45]. The following drugs belong to this group: midostaurin, lestaurtinib, tandutinib, sunitinib, cabozatinib, ponatinib, and ibrutinib [49,60]. The second-generation FLT3 inhibitors were developed after a number of unsatisfactory results of first-generation FLT3i related to their side effects. Successfully, the second-generation FLT3i demonstrates greater potency, specificity, and longer half-life, owing to which have less off-target effects than the first-generation agents. This group includes the following substances: quizartinib, gilteritinib, ponatinib, and crenolanib [45,49,58].

Among the first-generation and type 1 FLT3 inhibitors, distinguished ones include midostaurin and lestaurtinib, and first-generation type 2 includes sorafenib and tandutinib. Second-generation type 1 inhibitors include gilteritinib and crenolanib, and second-generation type 2 include quizartinib [45,49,61].

### 2.4. First-Generation FLT3 Inhibitors

The substances most commonly used in AML treatment belonging to this group are midostaurin (type 1) and sorafenib (type 2). Others also classified in this group include tandutinib, sunitinib, and lestaurtinib.

#### 2.4.1. Midostaurin

Midostaurin (*N*-benzoyl-staurosporine, formerly referred to as CGP41251 and PKC412) is a synthetic indolocarbazole, an orally available small molecule multi-targeting tyrosine kinase inhibitor that inhibits multiple kinases, including FLT3 which nowadays is utilized/used as an antineoplastic agent in treating acute myeloid leukemia with *FLT3* mutations.

Originally, it was developed as a pan kinase C inhibitor (PKCi) stemming from *Streptomyces staurosporeus* bacterium to improve outcomes of staurosporine treatment [62,63]. Later, it was discovered to be active against multiple tyrosine kinase receptors like c-KIT, PDGFR, VEGFR, PKC-alpha, cyclin-dependent kinase 1 (CDK1), cellular Src kinase (SRC), Fgr, spleen tyrosine kinase (Syk), and the main subject of our research—FLT3 [29,34,35]. Midostaurin has been investigated in a few pathological conditions for its multiple kinase inhibition. As a previously mentioned PKC inhibitor, midostaurin has not demonstrated efficacy in early-phase studies as monotherapy in low-grade lymphoproliferative disorders and metastatic melanoma and as combination therapy in various solid tumors [64,65,66].

Midostaurin can be described as a first-generation, type 1 FLT3 inhibitor [45]. In combination with intensive chemotherapy, in April 2017, it was approved by the US FDA for the frontline treatment of adult patients with newly diagnosed *FLT3*-mutated AML, at the same time being the first drug to receive regulatory approval for AML treatment in the US since the year 2000 [67]. The decision was made based on the results of RATIFY (CALGB 10603)—a global, randomized, double-blind, placebo-controlled phase 3 trial carried out in 225 centers in 17 countries—that establish whether the addition of midostaurin for induction and consolidation, with subsequent 1 year of maintenance treatment, will improve OS of AML patients with *FLT3* mutation aged 18 to 59 years. Patients with *FLT3*–ITD and *FLT3*–TKD mutations were enrolled regardless of allele ratio [45,67].

Midostaurin is present in all three stages of the currently suggested frontline treatment regimen for *FLT3*-mutated acute myeloid leukemia submitted in the 2022 ELN recommendations. For newly diagnosed AML with *FLT3* mutations, it has become standard to add midostaurin (dosage 50 mg q12h PO d8–21) to induction therapy in which the foundation is composed of anthracyclines (e.g., daunorubicin, idarubicin) and cytarabine in the “7+3” scheme. Alternative options for intensive chemotherapy may include fludarabine, granulocyte colony-stimulating factor and idarubicin (FLAG–IDA), and mitoxantrone-based cytarabine regimens. Consolidation therapy for AML *FLT3*-mutated relies on 3–4 cycles of IDAC 1000–1500 mg/m^2^ IV (500–1000 mg/m^2^ if ≥60 y old) over 3 h q12h d1-3 plus midostaurin 50 mg q12h PO d8–21 (in all cycles). The third phase of the *FLT3*-mutated AML treatment regimen is maintenance therapy, which the FDA defines as a prolonged but time-limited course of treatment that is usually less toxic, given after the achievement of CR has been reached to reduce the risk of relapse. It consists of dosing 50 mg midostaurin q12h p.o. d1–28, q4 wk, over 12 cycles [51].

In addition to daunorubicin–cytarabine induction therapy and high-dose cytarabine consolidation in adults <60 years, midostaurin improved 4-year OS by 7% (44.3 to 51.4%). Studies on maintenance showed it to be well tolerated; however, its efficacy needs to be further studied because the value of adding maintenance therapy is still uncertain [68]. In a prospective, non-randomized study, midostaurin also demonstrated a beneficial effect in patients up to 70 years of age compared with a historical, matched cohort [69].

Midostaurin, as other FLT3is, is generally well tolerated, especially when we compare its safety profile to classical chemotherapy agents or even with more recent immunotherapies, which allows their use in both fit and unfit AML patients, alone or combined with other drugs [70]. However, due to its wide scope of action and low selectivity, midostaurin exhibits many side, off-target effects. Among the most common adverse reactions reported in single-agent trials are nausea and vomiting, in combination trials—cytopenias. Also, in addition to expected hematologic toxicities, gastrointestinal toxicities seem to be increased compared to demethylating agents alone [63]. Other common side effects include fatigue, myalgia, arthralgia, fever, diarrhea, abdominal pain, dizziness, headache, hypotension, cough, and stomatitis. Potentially severe side effects include febrile neutropenia and sepsis, interstitial lung disease, and embryo-fetal toxicity. Midostaurin is metabolized in the liver predominantly by the cytochrome P450 system (mainly CYP3A4) and is vulnerable to interactions with inhibitors or inducers of the microsomal enzyme system, as well as potent CYP3A4 modulators should be avoided in patients receiving midostaurin [71].

#### 2.4.2. Sorafenib

Sorafenib (Nexavar) is an orally administered kinase inhibitor that targets both tumor cell proliferation and angiogenesis. Initially, it was developed as an inhibitor of Raf-1, a member of the Raf/MEK/ERK signaling pathway. After that, sorafenib was found to have activity against B-Raf, VEGFR-2, platelet-derived growth factor receptor (PDGFR), FLT3, and stem cell growth factor (c-Kit) [72]. Regarding FLT3i classification, sorafenib belongs to 1st generation type 2 FLT3i, which means it is only active against *FLT3*–ITD AML. Currently, sorafenib is approved for hepatocellular carcinoma and renal cell carcinoma therapy; however, it is often used off-label in AML treatment, most notably after HSCT, in numerous sites [61].

According to an open-label, multicentre, randomized phase 3 trial conducted by Xuan L. et al., [73] sorafenib maintenance following transplantation has the potential to decrease relapse rates. Additionally, it is well tolerated in patients with *FLT3*–ITD AML undergoing allogeneic hematopoietic stem cell transplantation. The annual relapse rate varied from 7% to 0% in patients who received sorafenib, while it ranged from 24.5% to 5% in patients assigned to the control group. The incidence of relapse significantly improved in almost all subgroups of patients who received sorafenib maintenance therapy after transplantation compared with those who did not receive maintenance therapy. Non-recurrence-related mortality showed no significant difference between the two groups. Thus, overall survival and leukemia-free survival showed a better outcome in the sorafenib maintenance therapy group compared with the control group. Measures of tolerability, such as the overall incidence of grade 3 and 4 adverse events at 210 days post-transplant, were similar in both treatment groups. Similar results and conclusions were reached in other independent studies [73,74].

Such results lead us to think that this approach may be a possible therapeutic option for patients with *FLT3*–ITD AML [73]. Perhaps this applies not only to the adult group of patients because similar conclusions regarding the treatment of pediatric patients with *FLT3*-mutated AML are provided by the JA Pollard et al. study, which explores the subject of treatment with sorafenib added to standard chemotherapy [75].

Unfortunately, quite a few trials of first-generation FLT3i were obstructed due to their off-target effects, non-optimal potency, low tolerability, poor clinical benefit in monotherapy, or resistance mechanisms [49].

### 2.5. Second-Generation FLT3 Inhibitors

#### 2.5.1. Gilteritinib

Gilteritinib is a second-generation type 1 tyrosine kinase inhibitor (TKI) that primarily targets FLT3 and AXL (an oncogenic tyrosine kinase) receptors [61,76]. In September 2018, it was approved in Japan for the treatment of R/R *FLT3*-mutated AML, and 2 months later, it was approved in the same indications by the FDA [76].

Concerning the mechanism of binding to the FLT3 kinase domain as an FLT3 type 1 inhibitor, gilteritinib acts as an ATP-competitive agent by interacting with DFG-in conformation of *FLT3*–TKD and forming two hydrogen bonds with amino acids Cys694 and Glu692. However, gilteritinib also shows some other interactions, especially with the gatekeeper residue F691, which is presumed to be one of the leading reasons for gilteritinib resistance, as enzymatically confirmed [77].

Several trials, including gilteritinib as monotherapy, were conducted, i.e., ADMIRAL or Chrysalis Trial.

The phase 3 ADMIRAL study demonstrated that gilteritinib therapy yielded significantly superior OS outcomes in patients with *FLT3*-mutated R/R AML compared to salvage chemotherapy (SC). The median OS for patients receiving gilteritinib was 9.3 months, whereas for those on salvage chemotherapy, it was 5.6 months. Additionally, the complete remission/complete remission with partial hematologic recovery (CR/CRh) rate was notably higher in the gilteritinib group (34%) than in the salvage chemotherapy group (15.5%).

Further analysis with a longer follow-up period revealed a reduction in adverse events among patients receiving gilteritinib treatment. Moreover, the estimated 2-year survival rate for the gilteritinib arm was 20.6%, whereas the corresponding rate in the salvage chemotherapy arm was 14.2% [78].

Interestingly, even in subgroups of patients who had previously undergone tyrosine kinase inhibitor treatment, gilteritinib therapy still led to higher response rates compared to the corresponding salvage chemotherapy cohort [79]. A retrospective analysis yielded real-world data pertaining to the utilization of gilteritinib in relapsed/refractory (R/R) patients who had received prior treatment with an FLT3 inhibitor. The findings revealed a noteworthy CRc rate of 48.7%, alongside a median OS of 7 months [80].

A phase 3 trial compared activity between azacitidine (AZA) + gilteritinib (GILT) and AZA alone in ND patients with *FLT3*-mutated AML unfit for intensive chemotherapy. The results demonstrated a significant difference in CRc rates between the two treatment arms, with patients receiving AZA + GILT showing a notably higher CRc rate of 58.1% compared to those treated with AZA alone, which yielded a CRc rate of 26.5%. However, despite the improved CRc rate, the OS did not differ significantly between the two cohorts. The safety profile of the AZA + GILT regimen was similar to the AZA alone [81].

#### 2.5.2. Quizartinib

Quizartinib is a highly potent second-generation type 2 FLT3 inhibitor with additional targeting of KIT and PDGFR isoforms, albeit with an affinity at least ten times lower than its primary FLT3 inhibition [82,83].

In 2019, Quizartinib was approved as an FLT3 inhibitor in Japan for the treatment of *FLT3*–ITD mutated R/R AML, and recently, on 20 July 2023, the FDA approved combination of quizartinib with a “7+3” standard chemotherapy [84]. In preclinical models, quizartinib showed activity in both *FLT3*–ITD mutated and *FLT3* wild-type line cells [85].

The phase 2 and 3 trials were designed to evaluate the efficacy and safety of the quizartinib monotherapy in *FLT3*–ITD mutated R/R AML patients. The CRc rate was 46–56%, and the median OS was 6.2 months in comparison to the median OS of 4.7 months in the salvage chemotherapy cohort. Moreover, the expected 1-year OS in quizartinib and salvage chemotherapy cohorts was 27% and 20%, respectively. The most common serious adverse events were febrile neutropenia (38%), AML progression (22%), pneumonia (12%), QTcF prolongation (10%), pyrexia (3%), and sepsis (2%) [86,87].

Phase 3 trial evaluating the efficacy of intensive chemotherapy in combination with quizartinib or placebo in AML patients with *FLT3*–ITD mutation presented similar CRc rates (71.6% vs. 64.9%, respectively) and *FLT3*–ITD MRD negativity status (24.6% vs. 21.4%, respectively). However, the median OS was significantly longer in patients receiving IC with gilteritinib than in IC with the placebo group (31.9 months vs. 15.1 months, respectively). Patients treated with quizartinib were, however, more likely to experience serious adverse events [83].

### 2.6. Mechanisms of FLT3 Inhibitors Resistance

**Figure 2:**
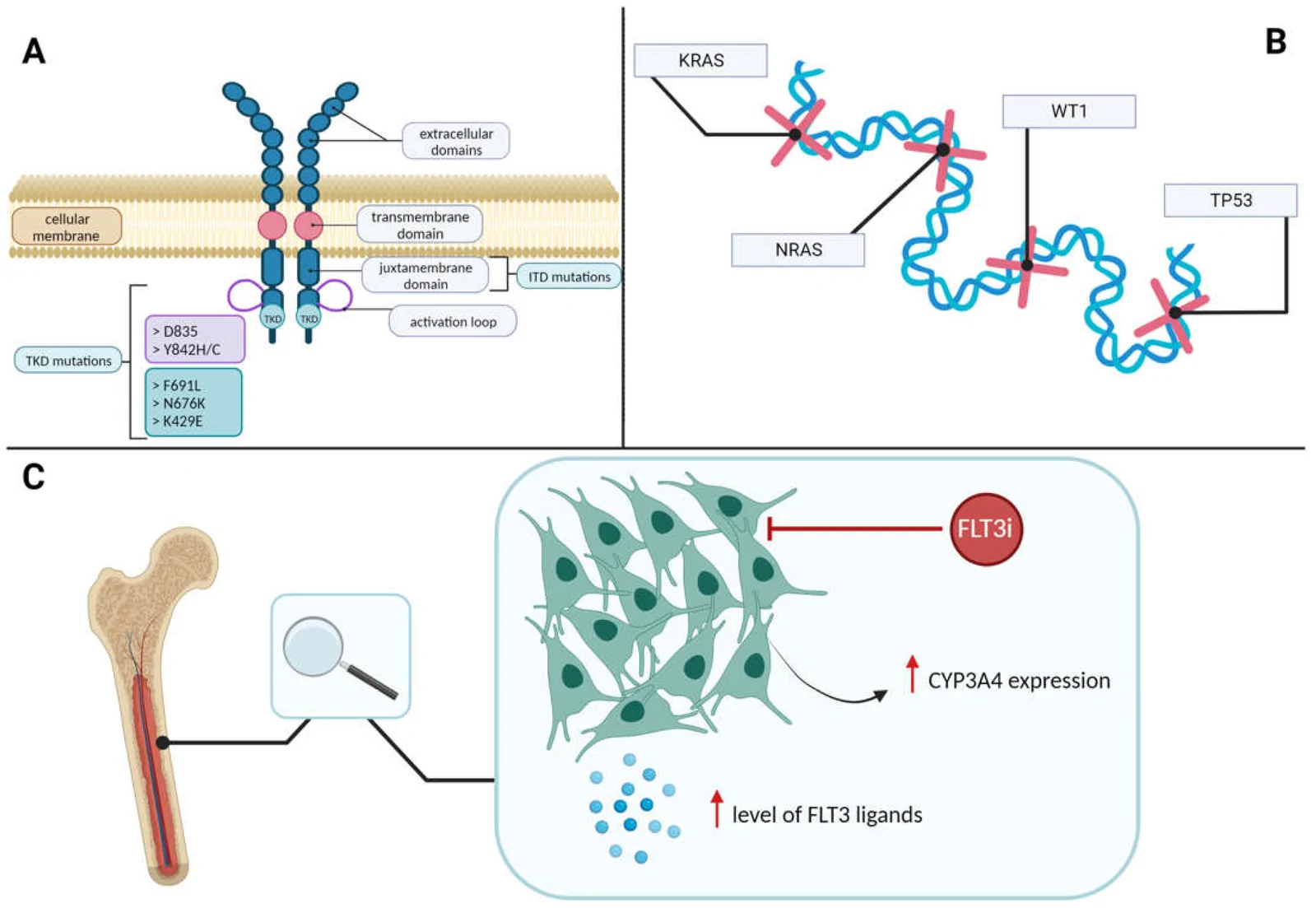
Mechanisms of resistance to FLT3 inhibitors. (**A**): Scheme of FLT3 kinase and occurring on-target mutations responsible for the emergence of FLT3i resistance. (**B**): Off-target mutations found to confer resistance to FLT3 inhibition. (**C**): Bone marrow microenvironment. Affected by treatment with FLT3i, stromal cells can secrete increased amounts of FLT3 ligands and upregulate CYP3A4, leading to rapid degradation of FLT3i. Created with BioRender.com (accessed on 17 August 2023).

The development of targeted therapies, including FLT3 inhibitors, has undeniably revolutionized the approach to AML treatment. However, even with therapies based on their use, limitations and challenges remain, particularly in terms of relapse and the emergence of treatment resistance. Thus, understanding the mechanisms underlying the resistance is of paramount importance due to its great clinical relevance. The current approach to the treatment of AML with *FLT3* mutations includes the use of midostaurin as part of initial chemotherapy, followed by gilteritinib as a stand-alone treatment for relapsed disease [51]. Patients often develop secondary mutations that are responsible for the emergence of resistance to these drugs, including mutations in *FLT3* itself [57]. When discussing the mechanisms of resistance that occur, two kinds of resistance should be mentioned: genetic and non-genetic. Also, primary resistance (refractory disease) and secondary resistance (relapsed disease) can be distinguished. Addressing the topic of genetic mechanisms, we have to mention that a series of mutations related to FLT3i resistance has already been identified—some of them as on-target mutations and some of them as off-target mutations. Among the first group of on-target mutations (Figure 2A), we should certainly note that *FLT3* F691L, known as a gatekeeper mutation situated within the active site of TKD, imparts resistance to all FLT3 inhibitors currently in clinical use [88,89]. In addition, *FLT3* N676K in the TKD domain has been shown to confer resistance to midostaurin—a well-known medicine used in frontline treatment in *FLT3*-mutated AML [90]—and the K429E mutation is associated with resistance to crenolanib [91]. On the other hand, we have mutations in the activation loop of FLT3, for example, *FLT3* D835 (D835F/V/Y) or *FLT3* Y842C/H, which result in resistance only to FLT3 type 2 inhibitors such as quizartinib or sorafenib [92,93]. The presence of secondary *FLT3* mutations is also thought to be one of the main factors contributing to the decline in rates of composite CR (CRc) in patients during sequential treatment with FLT3 inhibitors. Yet, Schmalbrock and colleagues’ comprehensive study to uncover the genetic causes of midostaurin resistance in *FLT3*–ITD-mutated AML found that only 11% of patients showed secondary *FLT3*–ITD mutations in relapsed or refractory AML. Instead, they identified the predominant mechanisms of resistance in their patient cohort as the proliferation of *FLT3*–ITD mutation-free subsets and the presence of mutations in signaling pathways downstream of *FLT3*. Referring to off-target mutations (Figure 2B), numerous studies utilizing advanced next-generation sequencing (NGS) techniques have revealed a relevant mechanism in forming resistance to FLT3 inhibitors. This mechanism involves the activation of alternative signaling pathways, such as PI3K/AKT/mTOR, RAS/RAF/MEK/ERK, JAK/STAT, and SRC family kinases, driven by non-target mutations in genes like *RAS/MAPK* pathway genes, *WT1*, and *TP53* [59,94,95,96,97].

On the subject of non-genetic causes of FLT3i resistance, we undoubtedly should refer to the overexpression of anti-apoptotic proteins, which is considered to be a critical mechanism of resistance to FLT3i. The study conducted by Yoshimoto et al. presented a mechanism of upregulation of MCL-1 anti-apoptotic protein in *FLT3*–ITD AML via STAT5 activation, making it a possible pathway for mediating the resistance [98]. Leukemic cells that have undergone malignant transformation seem to be more dependent on anti-apoptotic BCL-2 proteins compared to normal leukocytes. This occurs due to the stress induced by oncogenic agents and other cellular stresses associated with malignant transformation, triggering the activation of proapoptotic BH3-only proteins. Unless these proapoptotic proteins are counteracted by members of the anti-apoptotic family, leukemic cells that have undergone transformation may not be able to survive [99]. In cellular models reported by Kohl et al., it was observed that the heightened expression of BCL2 family proteins grants resistance to FLT3 inhibitors, allowing hematopoietic cells to elude programmed cell death [100]. Therefore, it can be concluded that this increased expression can be therapeutically counteracted by the use of BCL2 inhibitors, and it seems to be a promising therapeutic target. On the other hand, the Hunter et al. study noted that the high expression levels of P-glycoprotein (P-gp, a membrane efflux pump, which is recognized to have an anti-apoptotic function in cells with *FLT3*–ITDs) can reduce their sensitivity to FLT3 inhibitors and therefore P-gp expression should likewise be assessed in clinical trials of FLT3 inhibitors [101].

Another crucial factor that has to be addressed is the bone marrow microenvironment, otherwise known as the stem cell niche (Figure 2C), which plays a key role in enabling growth, survival, and the evolution of drug resistance in leukemic cells [89]. Bone marrow stromal cells (BMSCs) physiologically secrete FLT3 ligands. This growth factor has also been identified as a mediator of resistance to FLT3 inhibition, as evidenced by, for example, a study presented by Sato et al., which notes elevated levels of FLT3 ligand in patients who had relapsed compared to patients who had just been diagnosed with AML [102]. Furthermore, Chang et al. demonstrated that primary stromal cells within the bone marrow environment could facilitate the degradation of three different FLT3 inhibitors, sorafenib, quizartinib, and gilteritinib, by producing CYP3A4. This degradation resulted in reduced effectiveness of the FLT3i in vivo. The team also reported that this BMSC-mediated effect can be significantly reversed with clarithromycin, a clinically active, potent CYP3A4 inhibitor. This result suggests that combining FLT3 TKIs with CYP3A4 inhibitors could be a promising strategy for improving the activity of FLT3 TKIs [103].

### 2.7. Predictive Factors of Venetoclax and FLT3 Inhibitors

In the rapidly evolving landscape of AML treatment, the identification and understanding of specific biomarkers, co-occurring mutations, and their impact on responses have emerged as pivotal components in optimizing therapeutic strategies.

Preclinical data from a study conducted by Kivioja et al. showed that allelic ratio (AR) of *FLT3*–ITD mutation impacts the response to the FLT3 inhibitors. While low AR is associated with better response to non-specific TKI, patients with high AR respond more favorably to highly selective FLT3 inhibitor treatment. Moreover, the researchers found that measuring changes in *HLF* gene expression alongside the ITD–AR can improve the prediction of FLT3 inhibitors efficacy [104]. Another study regarding *FLT3*–ITD AML discovered that IL15 plays a role in mediating resistance to FLT3 inhibitors by increasing phosphorylation of ERK in FLT3i-resistant samples, but it can also be used as a biomarker predicting the response to targeted treatment with FLT3i [105]. Furthermore, it is suspected that a cytokine CCL5 also mediates resistance to FLT3 inhibitors and holds promise to become a valuable biomarker for predicting the efficacy of FLT3 inhibitor-based therapeutic strategy [106].

Co-occurring mutations can also impact the prognosis in *FLT3* mutated AML. Mutations in *CEBPA* and *NPM1* are associated with better outcomes, while mutations in *ASXL1* and *DNMT3A* predict worse outcomes [107].

Levels of BCL-2, BCL-XL, and MCL-1 proteins have been a well-known factor influencing the therapeutic response to venetoclax. Currently, recent advancements have emerged in this domain. It has been ascertained that the overexpression of *HOXA* and *HOXB*, gene families historically associated with a plethora of malignancies, was notably prevalent in venetoclax-sensitive cellular populations. Conversely, cells exhibiting resistance displayed diminished levels of *HOX* gene expression. Furthermore, an elevated mRNA expression of *B2M* (β2-microglobulin) has been identified as a potential correlate of resistance to venetoclax [108]. Additionally, high expression of *S100A8* and *S100A9* may be connected with resistance to venetoclax and can potentially be used to predict responses. Moreover, the authors suggest that the addition of BET inhibitor OTX-015 may help overcome the venetoclax resistance [109]. Resistance to venetoclax with azacitidine therapy is also more anticipated in patients with monocytic AML, possibly due to MCL-1 mediated regulation of OXPHOS [110].

Co-mutations are also an important factor influencing the prognosis of response to venetoclax. Mutations within genes such as *WT1, IDH1/2, SRSF2, NPM1,* and *ZRSR2* have been associated with more favorable responses to venetoclax. In contrast, mutations within *PTPN11*, *FLT3*–ITD, *KRAS/NRAS,* or *TP53* are linked with resistance to BCL-2 inhibitors [108,109,111].

As a method of assessing the possible effects of venetoclax treatment, Bhatt et al. demonstrated that with dynamic BH3 profiling, it is possible to identify drugs that are capable of overcoming resistance to BH3-mimetics in myeloblasts, exemplified by FLT3 inhibitors or SMAC mimetics. Furthermore, the authors recommend the administration of both BCL-2 and MCL-1 inhibitors simultaneously to help overcome the resistance and improve efficacy [109,112].

### 2.8. BCL-2 and FLT3 Inhibitors

**Figure 3:**
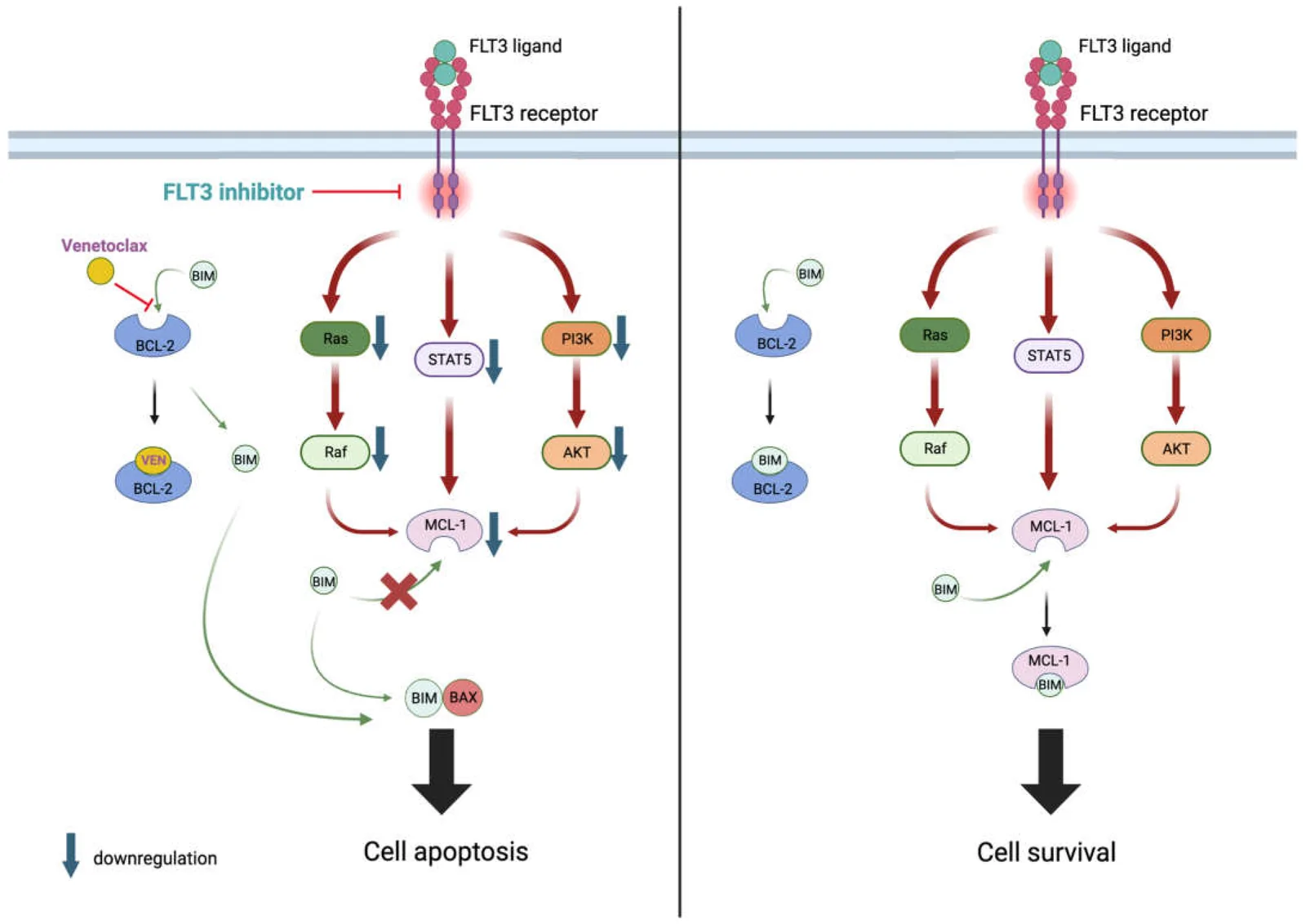
The mechanism of cell apoptosis by the combination of venetoclax and FLT3 inhibitors. The downregulation of MCL-1 prevents the binding of displaced BIM to MCL-1, therefore mediating the apoptosis. Created with BioRender.com (accessed on 17 August 2023).

In 2013, Andrew J Souers introduced ABT-199/venetoclax to the world, and it held promise for robust effects in early publications. Despite the disappointing efficacy of 19% in AML monotherapy, further research resulted in treatment regimens incorporating venetoclax, which are now the standard of care [15,113]. However, inhibiting the BCL-2 protein exclusively appears to be insufficient and leaves room for improvement. Venetoclax induces apoptosis by separating BIM from BCL-2. However, the freed BIM can be seized by MCL-1, thus preventing cell apoptosis. This mechanism gives the rationale for seeking agents capable of downregulating the MCL-1 level [114]. Data from preclinical studies suggest that FLT3 inhibition modulates MCL-1 directly through suppression of STAT5 and indirectly through suppression of RAS–MAPK and PI3K–AKT simultaneously (Figure 3) [115]. Studies investigating the molecular mechanisms underlying the effects of the combination of venetoclax and FLT3 inhibitors discovered that the simultaneous use of the two substances resulted in a decline in phosphorylated ERK (pERK) expression, thereby preventing the development of resistance. Moreover, the downregulation of MCL-1 mediated by FLT3 inhibitors enhances the apoptotic activity of venetoclax [116,117]. Preclinical data further demonstrated that, in *FLT3*–ITD cell lines, the administration of quizartinib led to a notable reduction of approximately 20–40% in BCL-XL protein levels and a more substantial decrease of about 60–80% in MCL-1 protein levels. The concomitant inhibition of the BCL-2 protein by venetoclax results in the simultaneous targeting of all three anti-apoptotic proteins, thereby yielding in vivo anti-tumor activity achievable with clinically attainable doses [118]. Additionally, the synergistic effect of venetoclax with gilteritinib or midostaurin was also observed in the *FLT3*-WT cells, probably due to the inhibition of other kinases by midostaurin and the affinity to AXL by gilteritinib [117]. Janssen et al. found that *FLT3*-WT cells can exhibit high FLT3 signaling, which is associated with resistance to venetoclax–azacitidine. Notably, among the compounds investigated for synergistic interactions with venetoclax, gilteritinib exhibited promising outcomes. This combination led to a significant reduction in the levels of pERK, pGSK3A/B, and pMCL-1 T163 and induced S159 phosphorylation, which was associated with proteasomal degradation of MCL-1, which mediates resistance to venetoclax [119].

Promising investigations into the synergy of BCL-2i and FLT3i have instigated the development of innovative therapeutic approaches. A notable instance is the research conducted by Fang et al., wherein they explored the combinatory potential of lisaftoclax (APG-2575), a novel BCL-2 inhibitor, and olverembatinib (HQP1351), an innovative BCR-ABL1 tyrosine kinase inhibitor that exhibits potent inhibitory activity against FLT3, fibroblast growth factor receptor, KIT, and various other oncogenic kinases. This investigation was conducted using preclinical models of *FLT3*–ITD AML. Results showed a significant reduction of tumor burden, thereby underscoring the promising clinical prospects for expanding the diversity of customized therapeutic options [120].

The lack of approved targeted drugs in patients with treatment-naïve AML harboring the *FLT3* mutation and unsuitable for chemotherapy and the low efficacy of single-agent therapy with short remission times in patients with R/R AML have prompted the search for additional effective treatments. Studies incorporating venetoclax into treatment with hypomethylating agents observed promising results [24,121].

In the treatment-naïve AML setting, patients treated with venetoclax and azacitidine achieved high remission rates, with a CR/CRi rate of 66.7%. In subgroups of *FLT3*–ITD and *FLT3*–TKD, the CR/CRi rates were 63.3% and 76.9%, respectively. Concurrent mutations of *FLT3*–ITD and *NPM1* were associated with a CR/CRi rate of 70%. The median OS was longer in patients with *FLT3*–TKD and reached 19.2 months compared to 9.9 months in the *FLT3*–ITD subgroup. Despite the high remission rates, the benefit in survival was observed only in the *FLT3*–TKD subgroup [24]. However, in other studies, CR/CRi rates in *FLT3*mut patients ranged from 40% to 94%, showcasing differences in response rates across studied groups [20,71,122].

In the relapsed/refractory AML setting, patients with the *FLT3* mutation achieved a weaker response to treatment than in the newly diagnosed setting. The CR/CRi rate was 42%, and the median OS was 8.3 months [121].

It is possible that mutations such as *TET2* or *ASXL1* positively influence the response to venetoclax. A surprising observation is the poor response in patients with a concomitant *IDH* mutation, which is generally associated with a good response to BCL-2 inhibitor treatment [20,121]. In addition, the co-mutation of *NPM1* and *DNMT3A* with the presence of an *FLT3* mutation in a group of 9 patients was associated with a modified composite complete response (mCRc) rate of 100% [52]. Contrary to these observations, a 2016 article suggests that the combination of these mutations is associated with significantly lower overall survival [123]. However, this study was conducted prior to the addition of venetoclax to treatment regimens, which may explain the change in response.

Another viable prognostic tool is MRD assessment. Studies show that MRD status correlates with treatment response. MRD-negative status in intermediate and low-risk patients receiving DEC10–VEN treatment is associated with significantly longer OS. In addition, based on the MRD status, the patient can be reclassified in the risk group from favorable to intermediate risk and vice versa [51,124].

Despite the fact that the implementation of venetoclax into treatment regimens resulted in improved outcomes, the researchers pursued the search for an optimal treatment method. In 2018, Daver et al. [52] conducted a clinical trial assessing the safety and efficacy of gilteritinib and venetoclax combination in patients with R/R AML. Out of the 61 enrolled patients, 56 harbored *FLT3* mutation. mCRc ratio was 75%, and the median OS was 10 months in participants with mutated *FLT3*. Data regarding the survival of patients who underwent alloSCT after the venetoclax/gilteritinib therapy are particularly encouraging. With a median follow-up time of 17.5 months, about 60% of patients who achieved this treatment were alive. Nonetheless, the adverse effects have to be taken into consideration. 97% of patients experienced grade 3/4 adverse effects, most commonly febrile neutropenia and pneumonia. The adverse effects caused venetoclax or gilteritinib interruptions in 51% of patients [52].

Following the encouraging results with the combination of venetoclax and gilteritinib, Maiti et al. conducted a study testing a triple combination of 20 mg/m^2^ IV decitabine, 400 mg PO venetoclax and the FLT3i (Table 1). In the ND setting, the CRc rate was 92%. Among patients that responded, 56% achieved the MRD-negativity by FCM and 91% by PCR/NGS. In the R/R setting, the CRc rate was 62%, and the MRD-negativity rate was 63% by FCM and 100% by PCR/NGS. The most frequent grade 3/4 adverse effects were febrile neutropenia (40%), infections with grade 3/4 neutropenia (36%), and tumor lysis syndrome (12%). The median follow-up in this study was 14.5 months, and the median OS was NR in ND patients and 6.8 months in R/R patients. The 18-month progression-free survival was similar in both groups, 59% in ND patients and 58% in R/R patients. The 2-year OS in patients who underwent HSCT for ND and R/R patients was 100% and 53%, respectively [125].

Yilmaz et al. [126] presented a retrospective study comparing the effects of triplet and doublet therapy in patients with mutated *FLT3* AML who were ineligible for intensive chemotherapy. In the triplet arm, the regimen included either 20 mg/m^2^ IV decitabine or 75 mg/m^2^ IV/SC azacitidine as a low-intensity chemotherapy backbone. In addition, each patient received gilteritinib, sorafenib, quizartinib, or midostaurin as an FLT3 inhibitor and venetoclax at a dose of 400 mg. The CR/CRi rates for patients treated with a triplet regimen were 93% vs. 73% in the doublet arm. MRD negativity measured by MFC was also significantly higher in the triplet group (83% vs. 38%). Median OS was not reached at a median follow-up of 12 months, and the 2-year OS rate was 70%. Surprisingly, the absolute number of patients with ANC > 500/mm^3^ and platelet count >50,000/mcL by day 42 from the start of therapy was higher in the arm treated with a triplet, probably as a result of the more than doubled CR rate with the triplet [126].

Further results presented by Yilmaz et al. show the efficacy and safety profile of the combination of DEC + VEN + QUIZ in both newly diagnosed and R/R settings. All patients received 10 days of 20 mg/m^2^ decitabine in cycle 1. Venetoclax was administered in a 400 mg dose for 14 or 21 days, depending on a bone marrow blast count. Quizartinib dose was 30 or 40 mg daily. Of 23 R/R patients, 78% achieved CR/CRi. 5 out of 18 responders achieved MRD negativity by MFC. The most common grade 3/4 non-hematologic adverse events were pulmonary infections (42%) and neutropenic fever (30%). The median OS in the R/R AML group was 7.6 months, and the 1-year OS was 30%. In the ND setting, all patients responded (2 CR, 3 CRi), and 2 out of 4 were MRD negative by MFC. Their median OS was 14.5 months. Interestingly, resistance to triplet therapy was associated with the *RAS*/*MAPK* mutation. Full results of this study are anticipated (NCT03661307) [127].

A trial by Short et al. aims to test the combination of azacitidine, venetoclax, and gliteritinib in ND AML patients ineligible for intensive chemotherapy or patients with R/R AML. Participants in both arms harbored the *FLT3* mutation. The first cycle consisted of azacitidine 75 mg/m^2^ for 7 days, venetoclax 400 mg for 28 days, and gilteritinib 80–120 mg for 28 days. Of the 40 patients treated, 21 were ND, and 19 were R/R. In the ND cohort, all participants responded, and the CR rate was 95%. 81% were MRD negative. The 6-month OS rate is 95%, and the estimated 1-year OS rate is 80%, with a median follow-up of 10.0 months. In the R/R group, 74% of participants responded, but only 37% achieved CR/CRi. The best response for the remaining 37% was MLFS (morphological leukemia-free state—defined as <5% blasts in the bone marrow with no blasts with Auer rods and no extramedullary disease). Of the 14 responding participants, 43% were MRD-negative. In this group, the median follow-up was 24.1 months, the median OS was 5.8 months, and the 1-year OS rate was 27%. Notably, the results were superior in patients who had not previously been exposed to HMA with venetoclax or gilteritinib (median OS: 10.5 months vs. 4.8 months; 1-year OS rate: 41% vs. 11%; *p* = 0.11) [128].

Chua et al. conducted a study to test the efficacy of a combination of midostaurin, venetoclax, and low-dose cytarabine in treatment-naïve patients over 60 years of age who were unsuitable for intensive chemotherapy and had non-adverse karyotype.

The ORR was 77.8%, and the CR rate was 44.4%. Median OS was not reached at a median follow-up of 18 months. Most of the participants were *FLT3*-WT. However, among those harboring the *FLT3*–ITD mutation, the combination of LDAC + VEN + MIDO resulted in the suppression of the mutation in all individuals.

Acquired resistance to this therapy was mediated by four pathways: *TP53* hotspot variants, *PPM1D* frameshift variants, *BAX* inactivating variant, and evolution of *KRAS* Q61 mutations.

Data comparing LDAC + VEN + MIDO to LDAC + VEN regimens will be presented from an ongoing phase 2 clinical trial (ACTRN12619001655134) [129].

Bergua-Burgues et al. [130] showed preliminary results of AZA or LDAC + VEN + QUIZ regimen in patients equal to or older than 60 years old with ND AML. In the second phase of this clinical trial, a total of 29 patients were successfully recruited, with 4 of them presenting *FLT3*-mutated AML. Among the enrolled participants, 14 were administered a treatment combination of LDAC + VEN + QUIZ, while the remaining 15 received AZA + VEN + QUIZ.

The ORR observed in both patient cohorts was found to be 54%. Throughout the course of the trial, the most frequently encountered adverse events included infections (*n* = 35) and gastrointestinal complications (*n* = 31). Notably, 48% of the patients had previously undergone treatment with AZA, demonstrating a subgroup with prior therapeutic interventions. As the study progresses and reaches its completion, further valuable data regarding follow-up information will become available, providing additional insights into the long-term effects and outcomes of the treatments under investigation (NCT04687761) [130].

Short et al. presented data from a subgroup analysis of patients with *FLT3*-mutated ND AML unfit for IC treated with a triplet regimen of AZA + VEN + GILT. A total of 30 participants were enrolled, all of whom responded positively. The CR/CRi was achieved by 96% (with a CR rate of 90%), while 4% achieved MLFS. Flow MRD negativity was observed in 87% of cases. The estimated RFS was 78%, and the 1-year OS was 86%, with a median follow-up time of 14.3 months. The outcomes were consistent irrespective of alloSCT performance. Furthermore, greater 1-year OS was noted in patients with *FLT3*–TKD mutation and those aged ≥75 years [131].

Abuasab et al. conducted a study on the implementation of venetoclax and gilteritinib to intensive chemotherapy consisting of cladribine, idarubicin, and cytarabine in patients with newly diagnosed *FLT3* mutated AML. The CLIA + VEN + GILT arm consisted of 8 patients, and 88% of them achieved CR/CRi. Of these, 71% were MRD-negative by MFC. The median OS was 22.4 months. The results of the triplet cohort were similar to the CLIA + GILT cohort, but the triplet regimen was associated with prolonged neutrophil and platelet recovery time [132].

A new study by Hua Jin et al. shows the efficacy and tolerability of combining venetoclax and azacitidine with homoharringtonine (VAH). In a subgroup of 19 participants with R/R AML harboring the *FLT3* mutation, an FLT3 inhibitor was added to the VAH regimen. 78.9% of patients achieved CRc, of which 58.8% achieved MRD-negative status. This study also showed high survivability; the 1-year OS was 73.7% [133].

## 3. Discussion

The implementation of venetoclax and FLT3 inhibitors in the treatment of AML has demonstrated promising results in terms of prognosis improvement and prolonging overall survival. Targeted therapy with midostaurin has led to the reclassification of AML with *FLT3* mutation into the intermediate-risk group, emphasizing the importance of MRD assessment in patient management. The incorporation of venetoclax and FLT3 inhibitors in triplet therapy regimens, often combined with low-intensity agents, has shown encouraging response rates and MRD negativity in both newly diagnosed and relapsed/refractory AML cases.

Triplet therapy exhibited significant progress in achieving complete remission and 1-year overall survival rates compared to doublet therapies, highlighting its potential as a more effective treatment option, especially in a population of newly diagnosed patients. However, the benefits of extended overall survival require further investigation on larger patient cohorts to validate the results.

While triplet therapy has shown promising outcomes, it also presented some challenges, such as increased myelosuppression and adverse events like neutropenic fever and pneumonia. Adjustments in drug dosing and treatment strategies may be necessary to manage these side effects effectively.

Furthermore, patients who have not been previously exposed to venetoclax or FLT3 inhibitors tend to respond better to treatment, indicating the potential for treatment-naïve patient populations.

Although the current findings are promising, the available data are still limited, warranting further research focusing on evaluating patient survival benefits and monitoring adverse effects. Continued investigation in phase 2 and 3 studies will be essential to optimize drug dosing and achieve the most favorable treatment outcomes.

In conclusion, the integration of venetoclax and FLT3 inhibitors into AML treatment regimens shows great potential for improving patient outcomes. With additional research and data, these targeted therapies may play a pivotal role in expanding the spectrum of treatment options for AML patients in the future.

## Figures and Tables

**Table 1:** Results of recent trials of triplet combination therapies.

Authors	Treatment Regimen	Phase of a Trial	Number of Participants	Median Age	Outcomes	Survivability
Abhishek Maiti et al. [125]	DEC + VEN + FLT3i	II	ND AML—12	70	CRc—92%	2-year OS—80%
			R/R AML—13	50	CRc—62%	mOS—6.8 months
Musa Yilmaz et al. [126]	LIC + VEN + FLT3i	retrospective	ND AML—27	69	CR/CRi—93%	mOS—NR with a median 12-month follow-up
Musa Yilmaz et al. [127]	DEC + VEN + QUIZ	I/II	ND AML—5	69	CR/CRi—100%	mOS—14.5 months
			R/R AML—23	-	CR/CRi—78%	mOS—7.6 months
Nicholas J. Short et al. [128]	AZA + VEN + GILT	I/II	ND AML—21	68	ORR—100%	6-month OS—95%, estimated 1-year OS—80%
			R/R AML—19	68	ORR—74%	mOS—5.8 months, 1-year OS—27%
Chong Chyn Chua et al. [129]	LDAC + VEN + MIDO	Ib/II	ND AML—18	77	ORR—77.8%	mOS—NR, with a median 18-months follow-up
Juan Miguel Bergua-Bergues et al. [130]	AZA + VEN + QUIZ or LDAC + VEN + QUIZ	I/II	ND AML—45	76.5	ORR—54%	unknown
Nicholas Short et al. [131]	AZA + VEN + GILT	II	ND AML—30	71	CR/CRi—96%	Estimated 1-year OS—86%
Tareq Abuasab et al. [132]	CLIA + VEN + GILT	II	ND AML—8	55	CR—88%	mOS—22.4 months

Abbreviations: DEC—Decitabine; VEN—Venetoclax; FLT3i—FLT3 inhibitor; LIC—Lower Intensity Chemotherapy; QUIZ—Quizartinib; AZA—Azacitidine; GILT—Gilteritinib; CLIA—Cladribine, Idarubicin, Cytarabine; LDAC—Low-Dose Cytarabine; MIDO—Midostaurin; ND—Newly Diagnosed; R/R—Relapsed/Refractory; CRc—Composite Complete Remission; CR/CRi—Complete Remission/Complete Remission with Incomplete Hematologic Recovery; ORR—Overall Response Rate; NR—Not Reached.
